# $D_s$-optimality in copula models

**DOI:** 10.1007/s10260-016-0375-6

**Published:** 2016-12-23

**Authors:** Elisa Perrone, Andreas Rappold, Werner G. Müller

**Affiliations:** 1 IST Austria, Am Campus 1, 3400 Klosterneuburg, Austria; 2 Johannes Kepler University of Linz, Altenberger Strasse 69, 4040 Linz, Austria

**Keywords:** $D_s$-optimality, Copula selection, Design discrimination, Stochastic dependence

## Abstract

Optimum experimental design theory has recently been extended for parameter estimation in copula models. The use of these models allows one to gain in flexibility by considering the model parameter set split into marginal and dependence parameters. However, this separation also leads to the natural issue of estimating only a subset of all model parameters. In this work, we treat this problem with the application of the $D_s$-optimality to copula models. First, we provide an extension of the corresponding equivalence theory. Then, we analyze a wide range of flexible copula models to highlight the usefulness of $D_s$-optimality in many possible scenarios. Finally, we discuss how the usage of the introduced design criterion also relates to the more general issue of copula selection and optimal design for model discrimination.

**Electronic supplementary material:**

The online version of this article (doi:10.1007/s10260-016-0375-6) contains supplementary material, which is available to authorized users.

## Introduction

Design optimization is generally largely employed in many applied fields as a convenient tool to improve information drawn from experiments. Recently, in Perrone and Müller (2016), the authors have extended the classical equivalence theory of *D*-optimality to a wider class of models for the usage of *copulas*, i.e. restrictions of joint probability distributions of random vectors with uniform margins on the unit interval [0, 1].

In particular situations, the interest of the experimenter is on the estimation of a meaningful subset of the model parameters. This analysis can be performed by applying $D_s$-optimality. Such a design criterion is particularly useful in designing experiments under assumption of copula models, where the marginal and the joint behavior of the phenomenon are modeled separately and are reflected by different model parameters.

Furthermore, for flexible copula models, maximizing the information on a subclass of dependence parameters also relates to one of the most important tasks in copula modeling: the choice of the specific copula to employ. This task is usually performed through the usage of omnibus goodness-of-fit tests that require minimum assumptions, for recent reviews see, e.g., Berg (2009), Genest et al. (2009), or Fermanian (2013). Other more specific avenues consist in applying graphical tools (Michiels and Schepper 2013) or information based criteria (Grønneberg and Hjort 2014). In fully parametric models, as considered in this paper, the latter can be formulated in terms of functions of the Fisher information matrix, which will allow us to generate optimal designs for copula model discrimination. As stated, developments of powerful goodness-of-fit tests and strategies to avoid the wrong choice of the dependence constitute a considerable part of the literature on copulas. The issue of model choice or discrimination is in principle also a well known part of (optimum) experimental design theory and several criteria (e.g., $D_s$-optimality, *T*-optimality, *KL*-optimality) have been proposed [see Dette and Titoff (2009), López-Fidalgo et al. (2007), Studden (1980) and Deldossi et al. (2016) for a special application to copula models].

In this work we first extend the general theory of $D_A$-optimality to copula models. Then, we present the usage of the $D_s$-criterion for various purposes including the discrimination between various classes of dependences and possible scenarios. This is motivated by the well known equivalence between test-based (*T*-optimality) and estimation-based ($D_s$-optimality) criteria for linear nested models differing by a scalar parameter [see, for instance, Fedorov and Khabarov (1986)]. However, we argue that design according to the latter criterion may be also useful in the nonnested case. Finally, we show through some examples possible real applications.

## Background

In this section, we summarize basic definitions and properties of copula functions and design of experiments. We also present the usage of design techniques for the introduced classes of statistical models.

### Statistical modeling via copulas

The problem of specifying a probability model for dependent random variables $Y_1$ and $Y_2$ can be simplified by expressing the corresponding 2-dimensional joint distribution ${\mathbf {F}}_{{Y_1}{Y_2}}$ in terms of its two margins $F_{Y_1}$ and $F_{Y_2}$, and an associated 2-copula (or dependence function) *C* defined as follows.

#### Definition 1

A *two-dimensional copula* (or *2-copula*) is a bivariate function $C: [0,1]\times [0,1] \longrightarrow [0,1]$ with the following properties:

1. for every $$u_1$$, $$u_2 \in [0,1]$$
  1 $$\begin{aligned} C(u_1,0) = 0, \; C(u_1, 1) = u_1, \; C(0,u_2) = 0, \; C(1,u_2) = u_2; \end{aligned}$$
2. for every $u_1$, $u_2$, $u_3$, $u_4 \in [0,1]$ such that $u_1 \le u_3$ and $u_2 \le u_4$,
  $$\begin{aligned} C(u_3,u_4) - C(u_3,u_2) - C(u_1,u_4) + C(u_1,u_2) \ge 0. \end{aligned}$$

The connection between copulas and cumulative joint probability distributions is stated in Sklar’s Theorem (Sklar 1959), which affirms that for every 2-dimensional joint distribution ${\mathbf {F}}_{{Y_1}{Y_2}}$ there exists a 2-copula *C*, defined as in Definition 1, such that

2 $$\begin{aligned} \mathbf {F}_{Y_1Y_2} (y_1,y_2) = C(F_{Y_1}(y_1), F_{Y_2}(y_2)) \end{aligned}$$

for all reals $y_1$, $y_2$. Moreover, if $F_{Y_1}$ and $F_{Y_2}$ are continuous, then *C* is unique; otherwise, *C* is uniquely defined on $\text {Range}(F_{Y_1})\times \text {Range}(F_{Y_2})$. Conversely, if *C* is a 2-copula and $F_{Y_1}$ and $F_{Y_2}$ are distribution functions, then the function $F_{Y_1Y_2}$ given by (2) is a joint distribution with marginals $F_{Y_1}$ and $F_{Y_2}$.

As a consequence of Sklar’s theorem, parametric families of copulas represent a powerful tool in statistics to describe the joint relationship between dependent random variables. The issue of selecting the appropriate dependence within an assumed true parametric copula family relates to the meaningful role played by the copula parameters, which correspond, for instance, to a specific measure of association for the modeled random variables. As a matter of fact, assuming $Y_1$ and $Y_2$ to be two continuous random variables whose copula is $C(\cdot ,\cdot ;\alpha _1)$, the measure of association Kendall’s $\tau$ directly relates to the expectation of the random variable $W = C(U,V;\alpha _1)$, and can be explicitly written as

3 $$\begin{aligned} \tau = 4 \iint \limits _{[0,1]^2} C(u,v;\alpha _1) d C(u,v;\alpha _1) - 1, \end{aligned}$$

with $U, \, V \sim \mathcal {U}([0,1])$. Therefore, the relation in Eq. (3) results in a correspondence between the copula parameter $\alpha _1$ and a fixed $\tau$ value (Nelsen 2006).

To make advantage of copulas in statistical modeling, several research efforts have been made to provide a variety of parametric families that reflect fundamental statistical properties of dependent random variables such as exchangeability, association measures, and tail dependences (Joe 2014; Durante and Sempi 2015).

First examples of classical copula families have been derived from well-known classes of joint distributions. This is the case of the elliptical copulas and extreme value copulas respectively obtained from elliptical and extreme value distributions. In addition, flexible parametric copula families can be constructed by considering any finite *convex linear combination* *C* of $k \in \mathbb {N}$ 2-copulas $C_i$, with $i=1,\cdots , k$.

Other fundamental classes of copulas have been derived from mathematical functionals. A notable example of such a class is the family of Archimedean copulas (Genest and Mackay 1986; McNeil and Nešlehová 2009), which relate to the notion of triangular norms (Klement et al. 2000). Archimedean copulas have become very popular due to their interesting analytic properties which make them tractable for inferential purposes (Genest et al. 2011). Although Archimedean copulas represent a commonly used tool for applications, they are not suitable to describe many real scenarios as they belong to the class of *exchangeable copulas* [see, for instance, Genest and Nešlehová (2013)].

Roughly speaking, exchangeable copulas are copulas which do not change under any permutations of their arguments, i.e., copula functions which are symmetric. On the one hand, this mathematical property is suitable to describe the joint behavior of *exchangeable random variables*, i.e., continuous random variables $Y_1$ and $Y_2$ such that the random vector $(Y_1, Y_2)$ has the same joint distribution of the random vector $(Y_2,Y_1)$. On the other hand it could represent a strong limitation in many cases where a causality relationship between the two random variables $Y_1$ and $Y_2$ is desirable. Possible ways of quantifying non-exchangeability in copula models have been provided in the literature (Klement and Mesiar 2006; Nelsen 2007).

Although some classes of bivariate copulas can directly deal with non-exchangeability (Capéraà et al. 2000; Charpentier et al. 2014; Klement et al. 2005; Baets et al. 2007), many other copulas largely used in modeling belong to the class of exchangeable ones. To make these families suitable to a wider range of real phenomena, a possibility is to apply transformations which commute exchangeable copulas into non-exchangeable ones (Durante 2007; Frees and Valdez 1998; Khoudraji 1995). As an example, we here present the Khoudraji’s asymmetrization described in Khoudraji (1995) which we use later on in this work. Specifically, a given exchangeable copula $C(\cdot ,\cdot ; \alpha _1)$, with parameter $\alpha _1$, can be modified into the copula $C=C(\cdot ,\cdot ;\alpha _1,\alpha _2,\alpha _3)$ defined, for every $(u,v)\in [0,1]^2$, by

4 $$\begin{aligned} C(u,v;\alpha _1,\alpha _2,\alpha _3) = u^{\alpha _2} v^{\alpha _3} C(u^{1-\alpha _2}, v^{1-\alpha _3};\alpha _1), \end{aligned}$$

where $\alpha _2,\;\alpha _3 \in [0,1]$. For $\alpha _2\ne \alpha _3$, *C* is non-exchangeable. The usage of such a transformation in the design framework has already been discussed in Durante and Perrone (2016). Another possible application will be presented in Sect. 4.

In the next subsection we introduce the theoretical framework of experimental design for copula models already developed in Perrone and Müller (2016).

### Design of experiments for copula models

Let $\mathbf {x}^T = (x_1, \ldots , x_r) \in \mathcal {X}$ be a vector of control variables, where $\mathcal {X} \subset \mathbb {R}^r$ is a compact set. The results of the observations and of the expectations in a regression experiment are the vectors

$$\begin{aligned} \mathbf {y}(\mathbf {x})= & {} (y_1(\mathbf {x}), y_2(\mathbf {x})),\\ \mathbf {E}[\mathbf {Y}(\mathbf {x})]= & {} \mathbf {E}[(Y_1,Y_2)] = \varvec{\eta }(\mathbf {x},\varvec{\beta }) = (\eta _1(\mathbf {x},\varvec{\beta }),\eta _2(\mathbf {x},\varvec{\beta })), \end{aligned}$$

where $\varvec{\beta }=(\beta _1, \ldots ,\beta _k)$ is a certain unknown parameter vector to be estimated and $\eta _i \; (i = 1,2)$ are known functions.

Let us call $F_{Y_i}(y_i(\mathbf {x}); \varvec{\beta })$ the cdf margins of each $Y_i$ for all $i\in \{1,2\}$ and $c_{\mathbf {Y}}(\mathbf {y}(\mathbf {x}); \varvec{\beta }, \varvec{\alpha })$ the joint probability density function of the random vector $\mathbf {Y}$, where $\varvec{\alpha }=({\alpha }_1,\ldots , {\alpha }_l)$ are unknown (copula) parameters.

The aim of design theory is to quantify the amount of information on both sets of parameters $\varvec{\alpha }$ and $\varvec{\beta }$, respectively, from the regression experiment embodied in the Fisher Information Matrix (FIM).

The FIM $m(\mathbf {x}, \varvec{\gamma })$ for a single observation is a $(k +l) \times (k +l)$ matrix whose elements are

5 $$\begin{aligned} \mathbf {E} \left( - \dfrac{\partial ^2}{\partial \gamma _i \partial \gamma _j} \log [c_{\mathbf {Y}}(\mathbf {y}(\mathbf {x}); \varvec{\beta }, \varvec{\alpha })] \right) \end{aligned}$$

where $$\varvec{\gamma }=\{{\gamma }_1,\ldots ,{\gamma }_{k+l}\}=\{{\beta }_1,\ldots ,{\beta }_k,{\alpha }_1,, \ldots , {\alpha }_l\}$$ and

$$\begin{aligned} c_{\mathbf {Y}}(\mathbf {y}(\mathbf {x}); \varvec{\beta }, \varvec{\alpha })= \dfrac{\partial ^2}{\partial y_1 \partial y_2} C(F_{Y_1}(y_1(\mathbf {x}); \varvec{\beta }), F_{Y_2}(y_2(\mathbf {x}); \varvec{\beta });\varvec{\alpha }) \end{aligned}$$

is the joint density function represented through a copula *C* in accordance to Eq. (2).

For a concrete experiment with *N* independent observations at $n \le N$ support points $\mathbf {x_1},\ldots ,\mathbf {x_n}$, the corresponding information matrix $M(\xi , \varvec{\gamma })$ then is

$$\begin{aligned} M(\xi , \varvec{\gamma }) = \sum \limits _{i=1}^n w_i \; m(\mathbf {x_i},\varvec{\gamma }), \end{aligned}$$

where $w_i$ and $\xi$ are such that:

$$\begin{aligned} \sum \limits _{i=1}^n w_i = 1, \quad \xi = \left\{ \begin{array}{cccc} \mathbf {x_1} &{} \ldots &{} \mathbf {x_n} \\ w_1 &{} \ldots &{} w_n \end{array} \right\} . \end{aligned}$$

Approximate design theory is concerned with finding $\xi ^*(\varvec{\gamma })$ such that it maximizes some scalar function $\phi (M(\xi ,\varvec{\gamma }))$, the so-called design criterion. In Perrone and Müller (2016), the authors have developed the equivalence theory for the well known criterion of *D*-optimality, i.e. the criterion $\phi (M(\xi ,\varvec{\gamma })) = \log \det M(\xi ,\varvec{\gamma })$, if $M(\xi ,\varvec{\gamma })$ is non-singular. The equivalence theory presented in Perrone and Müller (2016) allows one to investigate the impact on the design of various model assumptions, where the dependence structure is reflected by different parametric copula families. A still neglected aspect is the role of the copula parameters as a source of information on the appropriate model to be used. In this work we focus on this aspect, which relates to the well-known design issue of model discrimination between rival models. First, we extend the equivalence theory for the $D_A$-criterion, and, as a consequence, for the $D_s$-criterion. Then, we apply $D_s$-optimality to flexible copula models and we interpret this approach as a possible way to find optimal designs which discriminate between rival models.

## $D_A$-, and $D_s$-optimality

In this section we provide the extension for the $D_A$-criterion of a Kiefer–Wolfowitz type equivalence theorem, assuming the dependence described by a copula model. We then illustrate the basic idea of the new approach through a motivating example already analyzed in Perrone and Müller (2016).

### Equivalence theory

In this work, we consider the case when the primary interest is in certain meaningful linear combination of parameters. Such combinations are element of the vector $A^T\varvec{\gamma }$, where $A^T$ is an $s \times (k+l)$ matrix of rank $s < (k+l)$. If $M(\xi , \varvec{\gamma })$ is non-singular, then the variance matrix of the least-square estimator of $A^T\varvec{\gamma }$ is proportional to $A^T \{ M(\xi , \varvec{\gamma }) \}^{-1} A$ and then a natural criterion, generalization of the *D*-optimality for this context, would be of maximizing $\log \det [A^T \{ M(\xi , \varvec{\gamma }) \}^{-1} A]^{-1}$. This criterion is called $D_A$ *-optimality* (Silvey 1980).

The following Theorem shows a generalization for the $D_A$-optimality of the Kiefer–Wolfowitz type equivalence theorem already proved in Perrone and Müller (2016) for *D*-optimality. We have omitted the proof as it is fully analogous.

#### Theorem 1

For a localized parameter vector $(\tilde{\varvec{\gamma }})$, the following properties are equivalent:

1. $$\xi ^*$$ is $$D_A$$-optimal;
2. for every $\mathbf {x} \in \mathcal {X}$, the next inequality holds:
  $$\begin{aligned} \text { tr }[ M(\xi ^*, \tilde{\varvec{\gamma }})^{-1} A (A^T M(\xi ^*, \tilde{\varvec{\gamma }})^{-1} A)^{-1} A^T M(\xi ^*, \tilde{\varvec{\gamma }})^{-1} m(\mathbf {x}, \tilde{\varvec{\gamma }})]\le s; \end{aligned}$$
3. over all $\xi \in \Xi$, the design $\xi ^*$ minimizes the function
  $$\begin{aligned} \max \limits _{x \in \mathcal {X}}\text { tr }[M(\xi ^*, \tilde{\varvec{\gamma }})^{-1} A (A^T M(\xi ^*, \tilde{\varvec{\gamma }})^{-1} A)^{-1} A^T M(\xi ^*, \tilde{\varvec{\gamma }})^{-1} m(\mathbf {x}, \tilde{\varvec{\gamma }})], \end{aligned}$$
  where $\Xi$ denotes the set of all designs $\xi$.

Although we here extend the theory to the general case of $D_A$-optimality, in the following our interest is in the first $s < (k+l)$ parameters, only. In such a case, $M(\xi , \varvec{\gamma })$ can be written as:

$$\begin{aligned} M(\xi , \varvec{\gamma }) = \left( \begin{array}{cc} M_{11} &{}\quad M_{12} \\ M_{12}^T &{}\quad M_{22} \end{array} \right) , \end{aligned}$$

where $M_{11}$ is the $(s \times s)$ minor related to the estimated parameters. Therefore, the simplified criterion is to maximize the function $\phi _s (M(\xi , \varvec{\gamma })) = \log \det (M_{11} - M_{12}M_{22}^{-1}M_{12}^T)$, which is called $D_s$ *-optimality*. We now have

#### Corollary 1

$D_s$-optimality follows as a particular case of Theorem 1 by the choice $A^T = (I_s \; 0)$.

Given the characterization of Corollary 1, two designs $\xi$ and $\xi ^*$ can be compared by means of a ratio called $D_s$ *-Efficiency* defined as follows:

$$\begin{aligned} \left( \dfrac{\det [M_{11}(\xi , \tilde{\varvec{\gamma }}) - M_{12}(\xi , \tilde{\varvec{\gamma }})M_{22}^{-1}(\xi , \tilde{\varvec{\gamma }})M_{12}^T(\xi , \tilde{\varvec{\gamma }})]}{\det [ M_{11}(\xi ^*, \tilde{\varvec{\gamma }}) - M_{12}(\xi ^*, \tilde{\varvec{\gamma }})M_{22}^{-1}(\xi ^*, \tilde{\varvec{\gamma }})M_{12}^T(\xi ^*,\tilde{\varvec{\gamma }})]}\right) ^{1/s}. \end{aligned}$$

In the next section we will describe the usage of $D_s$-optimality through a simple example originally reported in Fedorov (1971).

### $D_s$-optimality for copula models: A motivating example

Let us assume that for each design point $x \in [0,1]$, we observe an independent pair of random variables $Y_1$ and $Y_2$, such that

$$\begin{aligned} E[Y_1(x)]= & {} \beta _1 + \beta _2 x + \beta _3 x^2 ,\\ E[Y_2(x)]= & {} \beta _4 x + \beta _5 x^3 + \beta _6 x^4. \end{aligned}$$

The model is then linear in the parameter vector $\varvec{\beta }$ and has dependence described by the product copula with Gaussian margins.

This example has already been generalized in Perrone and Müller (2016) where various dependences through copula functions have been introduced and the corresponding *D*-optimal designs have been computed. In order to illustrate the usage of $D_s$-optimality in this context, let us assume the dependence between $Y_1$ and $Y_2$ described by a Clayton copula with $\alpha _1 = 18$, corresponding to a Kendall’s $\tau$ [see Eq. (3)] value of 0.9.

Even though the low losses in *D*-efficiency reported in Perrone and Müller (2016) suggest that the impact of the assumed dependence is completely negligible, one might aim at verifying whether the information related to the dependence structure is only carried by the estimation of $\alpha _1$. Essentially, one might focus on the six marginal parameters entirely disregarding the estimation of the dependence parameter $\alpha _1$. This can be done in practice by applying the $D_s$-optimality to the parameter vector $\varvec{\beta }$.

**Fig. 1:**
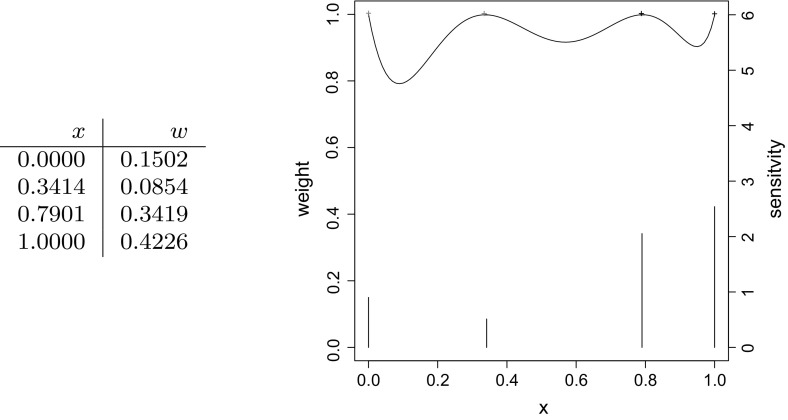
Design points (*first column*), weights (*second column*), sensitivity function (*continuous line*) and weights (*bars*) of the $D_s$-optimal design for $\beta _1,\ldots ,\beta _6$

Figure 1 shows the $D_s$-optimal design corresponding to this case. Comparing the *D*-optimal design of the product copula, assuming no dependence, with the $D_s$-optimal design for only the vector $\varvec{\beta }$, the loss in $D_s$-efficiency is of $8\%$. This shows that the dependence structure itself can substantially affect the design even if the dependence parameter $\alpha _1$ is ignored in the estimation.

In more complex models, a similar approach can be used to identify informative designs to specific properties of interest. In the following, we highlight the usefulness of flexible copula models through the application of the $D_s$-criterion to a subclass of meaningful model parameters. We construct in this way designs which better reflect the strength and the structure of a specific dependence and might be used to discriminate between classes of copulas.

## Bivariate binary case

We analyze an example with potential applications in clinical trials already examined in Denman et al. (2011) and Perrone and Müller (2016). We consider a bivariate binary response $(Y_{i1}, Y_{i2})$, $i=1, \ldots , n$ with four possible outcomes $\{ (0,0),(0,1),(1,0),(1,1)\}$ where 1 usually represents a success and 0 a failure (of, e.g., a drug treatment where $Y_1$ and $Y_2$ might be efficacy and toxicity). For a single observation denote the joint probabilities of $Y_1$ and $Y_2$ by $p_{y_1,y_2} = \mathbb {P} (Y_1 = y_1, Y_2 = y_2)$ for $(y_1,y_2 \in \{0,1\})$. Now, define6A particular case of the introduced model has already been analyzed in Heise and Myers (1996). In that work, the marginal probabilities of success are given by the models

7 $$\begin{aligned} \log \left( \dfrac{\pi _i}{1 - \pi _i} \right) = \beta _{i1} + \beta _{i2} x, \qquad i=1,2 \end{aligned}$$

with $x \in [0,10]$. As we are using nonlinear models the Fisher information will depend upon the unknown $\beta$ and thus we need to localize parameters at $\tilde{\varvec{\beta _1}}=(-1, 1)$ and $\tilde{\varvec{\beta _2}}=(-2, 0.5)$.

Let us now allow the strength of the dependence itself be dependent upon the regressor *x*. As in our context only positive associations make sense we consider in the following the corresponding Kendall’s $\tau$ modeled by a logistic:

$$\begin{aligned} \tau (x, \alpha _1) = \dfrac{e^{\alpha _1 x - c}}{1 + e^ {\alpha _1 x - c}}, \end{aligned}$$

where *c* is a constant chosen such that $\tau$ takes values in $[\epsilon ,1]$ for $\alpha _1 \in [0,1]$. For our computations we choose $\epsilon =0.05$ and we select three values for $\alpha _1$ such that the $\tau$ ranges are $I_1 = [0.05,0.3], \; I_2= [0.05,0.9]$, and $I_3= [0.05,0.95]$.

Then, using the relationship from Eq. (3) that associates the Kendall’s $\tau$ with the copula parameter, we model $p_{11}$ by pair convex combinations of Joe, Frank, Clayton, and Gumbel copulas by linking the two copulas $C_1$ and $C_2$ at the same $\tau$ values through the functions $h_1$ and $h_2$:

$$\begin{aligned} C(\pi _1,\pi _2; \alpha _1, \alpha _2) = \alpha _2 C_1(\pi _1,\pi _2; \; h_1(x,\alpha _1)) + (1-\alpha _2) C_2(\pi _1,\pi _2; \, h_2(x,\alpha _1)). \end{aligned}$$

Notice that the construction is more general and any convex combination of standard copulas from the R package ‘copula’ can be considered through the package ‘docopulae’ (Rappold 2015).

**Fig. 2:**
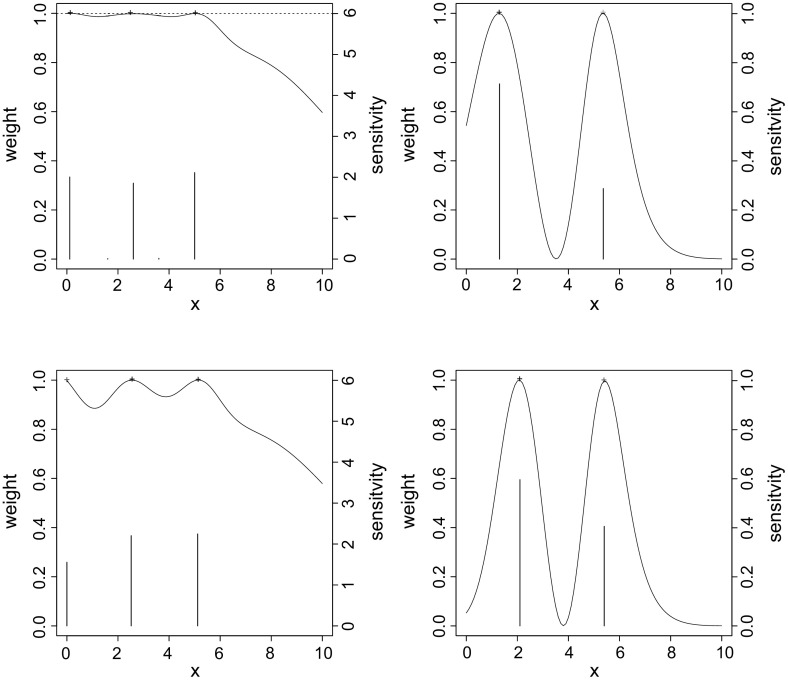
Sensitivity functions (*continuous lines*) and weights (*bars*) for *D*-optimal (*left column*) and $D_s$-optimal (*right column*) designs for Clayton-Gumbel (*first line*) and Frank-Gumbel (*second line*) with $\tau \in I_2=[0.05,0.9]$ and $\alpha _2=0.5$

In this model, the impact of the dependence structure and the association level is reflected by two different parameters, as the $\alpha _1$ parameter is only related to the measure of association Kendall’s $\tau$, while the $\alpha _2$ parameter is strictly related to the structure of the dependence. Therefore, applying the $D_s$-criterion on $\alpha _2$, we find a design for discriminating against the encompassing model. Evaluating at a local guess of $\tilde{\alpha }_2=0.5$, symmetry considerations lead us to believe that in this specific model we will also find good designs for discriminating between the two copulas considered.

We compare the design obtained for different $\tau$ intervals and localized values for $\alpha _2$ with the *D*-optimal design obtained for the same localized values (Fig. 2). Analyzing the rather high losses in $D_s$-efficiency reported in Table 1, it shows that the *D*-criterion alone is not sufficient when we require information about the structure of the model.

In this scenario, an interesting question is whether the obtained $D_s$-optimal designs are robust with respect to the initial model assumptions. To analyze this aspect, we computed the $D_s$-efficiencies for cross-comparisons of $D_s$-optimal designs. In Table 2, the results for $\tau \in I_2$ and $\tilde{\alpha }_2 = 0.5$ are reported (see Fig. 2, also). Looking at the table, one can notice that the losses correspondent to the assumed combination Clayton-Gumbel are in general lower, not exceeding $16\%$. This means that such a combination provides good results in order to discriminate between all the considered dependences. Further studies in this direction would lead to the development of new design techniques to construct robust and stable designs for discrimination between various classes of dependences.

**Table 1:** Losses in $D_s$-efficiency in percent for $I_1 = [0.05,0.3], \; I_2= [0.05,0.9]$, and $I_3= [0.05,0.95]$

$\tilde{\alpha }_2$	$\tau \in I_1$	$\tau \in I_2$	$\tau \in I_3$	$\tau \in I_1$	$\tau \in I_2$	$\tau \in I_3$
Joe–Frank				Clayton–Gumbel		
0.1	34.94	38.80	41.37	49.85	49.45	45.10
0.5	42.36	38.20	41.83	43.65	39.27	39.03
0.9	55.11	47.23	44.15	37.87	34.65	37.78
Joe–Clayton				Frank–Gumbel		
0.1	35.92	36.35	39.01	47.13	48.29	46.17
0.5	45.37	43.17	45.53	37.65	34.41	34.37
0.9	49.92	48.72	45.36	38.51	34.19	36.26

**Table 2:** Losses in $D_s$-efficiency in percent for $\tau \in I_2$ and $\tilde{\alpha }_2=0.5$ by comparing the true copula model with the assumed one

True copula	Assumed copula			
	C–G	F–G	J–C	J–F
Clayton–Gumbel (C–G)	0.00	28.44	7.43	19.07
Frank–Gumbel (F–G)	16.09	0.00	30.17	19.51
Joe–Clayton (J–C)	4.25	34.27	0.00	13.51
Joe–Frank (J–F)	15.13	13.97	9.52	0.00

## Bivariate discretized Weibull function

We now analyze an example originally reported in Kim and Flournoy (2015). In this example we construct original (nested) asymmetric copula models and we apply $D_s$-optimality to discriminate between symmetric and asymmetric scenarios. First investigations on the changes in the geometry of the *D*-optimal designs for such asymmetric copula models have been carried out in Durante and Perrone (2016), where a theoretical overview of exchangeability in the copula theory is also given.

We assume two dependent binary outcomes, *U* and *V*, for two system components, respectively. Considering 0 indicating no failure and 1 indicating failure, the outcome probabilities given a stress *x* can be written as:

$$\begin{aligned} p_{uv}(x, \varvec{\gamma }) = \mathbb {P}(U=u,V=v\mid x, \varvec{\gamma }), \end{aligned}$$

with $u,v \in \{ 0,1\}$ and where $\varvec{\gamma }$ denotes a vector of all the model parameters.

Let *Y* and *Z* denote the amount of damage on component 1 and component 2, respectively, and let $f(y,z\mid x,\varvec{\gamma })$ be the bivariate Weibull regression model. Suppose that failures are defined by dichotomizing damage measurements *Y* and *Z*:

8 $$\begin{aligned} \begin{array}{c} U = \left\{ \begin{array}{lll} 0 &{}\quad \text { (no failure for component 1),}&{}\quad \text {if}\, Y< \zeta _1, \\ 1 &{}\quad \text { (failure for component 1),}&{}\quad \text {otherwise}\end{array}\right. \\ \\ V = \left\{ \begin{array}{lll} 0 &{} \quad \text { (no failure for component 2),}&{}\quad \text {if}\, Z < \zeta _2, \\ 1 &{}\quad \text { (failure for component 2)},&{}\quad \text {otherwise}\end{array}\right. \end{array} \end{aligned}$$

where $\zeta _1$ and $\zeta _2$ are predetermined cut-off values. Then, the probabilities of success and failure are:

9 $$\begin{aligned} \begin{array}{l} p_{00} = \int _0^{\zeta _1} \int _0^{\zeta _2} f(y,z\mid x,\varvec{\gamma }) \,d y\,d z, \quad p_{01} = \int _0^{\zeta _1} \int _{\zeta _2}^{\infty } f(y,z\mid x,\varvec{\gamma }) \,d y\,d z, \\ \\ p_{10} = \int _{\zeta _1}^{\infty } \int _0^{\zeta _2} f(y,z\mid x,\varvec{\gamma }) \,d y\,d z, \quad p_{11} = \int _{\zeta _1}^{\infty } \int _{\zeta _2}^{\infty } f(y,z\mid x,\varvec{\gamma }) \,d y\,d z. \end{array} \end{aligned}$$

Now, considering $f(y,z\mid x,\varvec{\gamma })$ defined as follows:

$$\begin{aligned} f(y,z) = \left\{ \begin{array}{ll} \beta _1(\beta _3 + \beta _5) \kappa ^2 (yz)^{\kappa - 1} \text {exp}\{ -(\beta _3 + \beta _5) z^{\kappa } - (\beta _1 + \beta _2 - \beta _5) y^{\kappa }\} &{} \quad \text {for } 0< y< z< \infty ; \\ \beta _2(\beta _3 + \beta _4) \kappa ^2 (yz)^{\kappa - 1} \text {exp}\{ -(\beta _3 + \beta _4) y^{\kappa } - (\beta _1 + \beta _2 - \beta _4) z^{\kappa }\} &{}\quad \text {for } 0< z< y< \infty ; \\ \beta _3 \kappa (y)^{\kappa - 1} \text {exp}\{ -(\beta _1 + \beta _2 + \beta _3)\} &{} \quad \text {for } 0< y = z < \infty . \end{array}\right. \end{aligned}$$

The marginal survival functions of the bivariate Weibull density are weighted univariate Weibull survival functions:

$$\begin{aligned} \mathbb {P}(Y \ge y)= & {} \dfrac{\beta _2}{\beta _1 + \beta _2 - \beta _4} \text {exp} \{ - (\beta _3 + \beta _4) y^{\kappa }\} \\&\quad + \left( 1- \dfrac{\beta _2}{\beta _1 + \beta _2 - \beta _4}\right) \text {exp} \{ - (\beta _1 + \beta _2 + \beta _3) y^{\kappa } \}\\ \mathbb {P}(Z \ge z)= & {} \dfrac{\beta _1}{\beta _1 + \beta _2 - \beta _5} \text {exp} \{ - (\beta _3 + \beta _5) z^{\kappa }\}\\&\quad + \left( 1- \dfrac{\beta _1}{\beta _1 + \beta _2 - \beta _5}\right) \text {exp} \{ - (\beta _1 + \beta _2 + \beta _3) z^{\kappa } \} \end{aligned}$$

In Kim and Flournoy (2015), the authors set $\zeta _1=0.8$ and $\zeta _2 = 0.7$. Moreover, they consider the following predictor functions:

10 $$\begin{aligned} \left\{ \begin{array}{l} -\log (\beta _3 + \beta _5) = \theta _0 + \theta _1 x , \\ -\log (\beta _3 + \beta _4) = \theta _0 + \theta _2 x , \\ -\log (\beta _1 + \beta _2 + \beta _3) = \theta _0 + \theta _3 x. \end{array}\right. \end{aligned}$$

with $$x\in [0,1]$$.

In Kim and Flournoy (2015) the asymmetry in the causality has been reflected by different cut points, e.g., unequal values for $\zeta _1$ and $\zeta _2$, and different initial failure rates $\beta _1$ and $\beta _2$ as well as different coefficients $\theta _1$ and $\theta _2$ of the predictor.

**Fig. 3:**
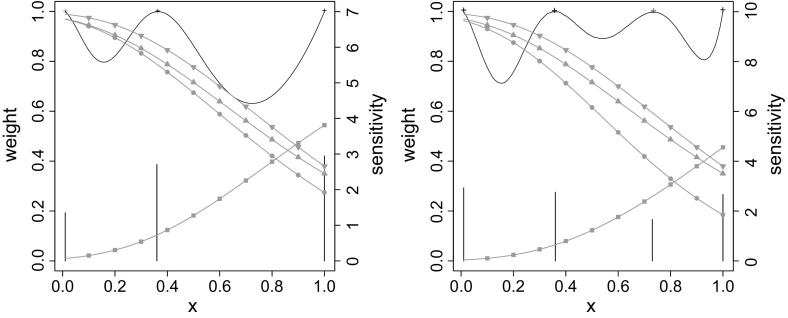
Sensitivity functions (*continuous lines*) and design weights (*bars*) of the *D*-optimal design for the Weibull case as reported in Kim and Flournoy (2015) (*left*), and for asymmetric Clayton with $(\tilde{\alpha }_1,\tilde{\alpha }_2,\tilde{\alpha }_3)= (1.5,0.4,0)$ (*right*); *filled circle* $p_{00}$; *filled square* $p_{11}$; *filled inverted triangle* $p_{0.}$; *filled triangle* $p_{.0}$

In our example, we additionally allow asymmetry of the phenomenon to appear in the dependence structure. In particular, such an asymmetry is introduced through the transformation presented in Eq. (4), adding new parameters in the process.

**Table 3:** Losses in *D*-efficiency in percent for crossed comparison between the optimal designs found for the Weibull model as reported in Kim and Flournoy (2015) and all our models

True model	Assumed model			
	Weibull		Our models	
	min	max	min	max
Weibull	0.00	0.00	9.43	10.18
Our models	17.78	71.65	0.00	3.37

**Fig. 4:**
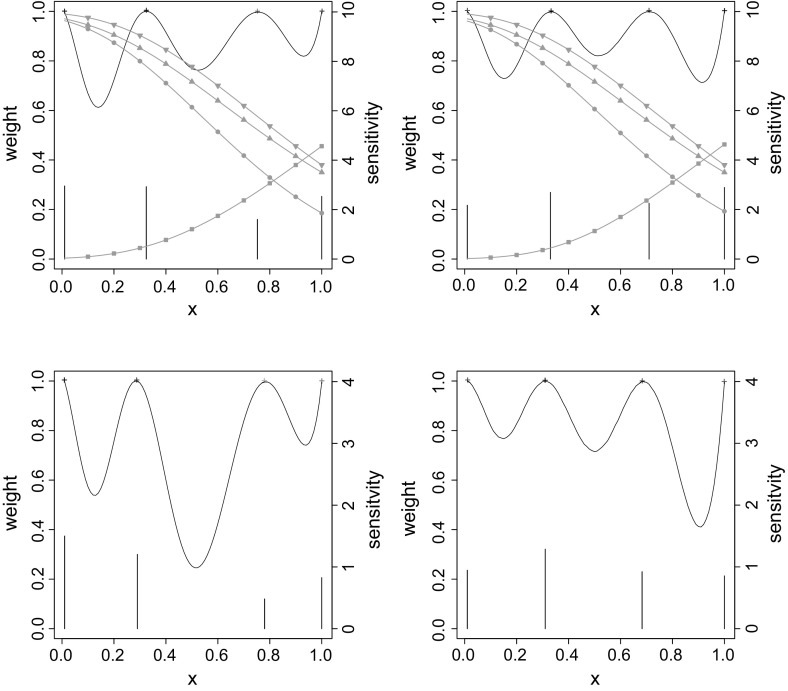
Sensitivity functions (*continuous lines*) and design weights (*bars*) of *D*-optimal designs (*first row*) and $D_s$-optimal designs (*second row*) for the Weibull case for asymmetric Clayton with $(\tilde{\alpha }_1,\tilde{\alpha }_2,\tilde{\alpha }_3)= (2,0.4,0.2)$ (*left column*), and for $(\tilde{\alpha }_1,\tilde{\alpha }_2,\tilde{\alpha }_3)= (3.6,0.6,0)$ (*right column*); *filled circle* $p_{00}$; *filled square* $p_{11}$; *filled inverted triangle* $p_{0.}$; *filled triangle* $p_{.0}$

Going into details, we introduce two parameters $\nu _1$, and $\nu _2$ such that the following is satisfied:

$$\begin{aligned} \left\{ \begin{array}{l} \theta _1 = \theta _2 + \nu _1,\\ \beta _1 = \beta _2 + \nu _2. \end{array}\right. \end{aligned}$$

The vector $(\nu _1, \nu _2)$ then quantifies the dissimilarity of the margins. For our study, we assume the joint dependence to be described by the asymmetric Clayton copula with three parameters $\alpha _1, \; \alpha _2$ and $\alpha _3$, constructed according to Eq. (4). In this context, we apply $D_s$-optimality to the parameters $\varvec{\mu } = (\nu _1, \nu _2, \alpha _2, \alpha _3)$ which denote the total asymmetry of the phenomenon, both from the marginals and the joint dependence. In such a way, we find designs which are more informative to the asymmetry and are then suitable to discriminate between exchangeable models and non-exchangeable ones. The used parameter setting corresponds to two Kendall’s tau values: 0.5 and 0.25, respectively. The initial values of the parameters ${\alpha }_1, {\alpha }_2,$ and ${\alpha }_3$ are the same as used in Durante and Perrone (2016), while the other parameter values are $\tilde{\theta }_0=-2,\; \tilde{\theta }_2=5,\; \tilde{\theta }_3=2,\; \tilde{\nu }_1=-1,\; \tilde{\nu }_2=0.1,\; \tilde{\beta }_2=0.2,$ and $\tilde{\kappa }=2$.

The *D*-optimal designs obtained spread weight to four design points, slightly differing in their distribution. Figure 3 shows a representative design for our model side by side with the *D*-optimal design for the Weibull case as reported in Kim and Flournoy (2015). The maximal and minimal values of the loss in *D*-efficiency by comparing the design reported in Kim and Flournoy (2015) and the *D*-optimal designs for our models are reported in Table 3. A full table with the losses of such comparison for each set of initial values of ${\alpha }_1, \; {\alpha }_2,$ and ${\alpha }_3$ is available in the supplementary material. The results suggest that in every case it would be advantageous to choose one of our models as generally more informative and robust.

We are now interested in verifying whether the *D*-optimal design is informative enough to discriminate between asymmetry and symmetry. To this aim, we compare $D_s$-optimal designs for $\varvec{\mu }$ to the corresponding *D*-optimal designs (Fig. 4). In this case, the loss in $D_s$-efficiency never exceeds $5\%$. In contrast to the binary case, such a result indicates that the *D*-optimal design is already quite adequate for discriminating between symmetric and asymmetric models.

## Conclusions

In this paper we extend the equivalence theory to allow the application of the $D_s$-optimality to copula models. In addition, we use the extended theory to embed the issue of the choice of the copula in the context of design discrimination. Specifically, we present a new methodology based on the usage of $D_s$-optimality to construct designs that discriminate between various dependences. Through some examples we highlight the strength of the proposed technique due to the usage of the copula properties. In particular, the proposed approach allows to check the robustness of the *D*-optimal design in the sense of discrimination and to construct more informative designs able to distinguish between classes of dependences.

All the shown results are obtained by the usage of the R package ‘docopulae’ (Rappold 2015). Although we here compare just a few possible dependences, the general construction is much wider. The R package ‘docopulae’ allows the interested reader to run designs assuming a broad variety of dependence structures. It then provides a strong computational tool to the usage of copula models in real applications.

In the future, we aim at generalizing other discrimination criteria such as *T*-optimality and *KL*-optimality to flexible copula models (Dette and Titoff 2009; Uciński and Bogacka 2005; López-Fidalgo et al. 2007). Furthermore, powerful compound criteria might be developed for such models [see, for instance, Atkinson (2008), Dette (1993) and Tommasi (2009)]. In addition, the construction of multistage design procedures that allow for discrimination and estimation might be of great interest in special applications such as clinical trial studies (Dragalin et al. 2008; Müller and Ponce De Leon 1996).

## Electronic supplementary material

Below is the link to the electronic supplementary material.

Supplementary material 1 (pdf 55 KB)
