# Supplementary material for: Ds-optimality in copula models
Source: Stat Methods Appt. 2016 Dec 23;26(3):403–18. doi: 10.1007/s10260-016-0375-6 (PMC5935038; doi:10.1007/s10260-016-0375-6)
Supplement: Supplementary file 1 — Supplementary material 1 (pdf 55 KB) [file 10260_2016_375_MOESM1_ESM.pdf]

	True Model								
Assumed Model	$(3.5, 0.2, 0.0)$	$(0.9, 0.2, 0.0)$	$(11.0, 0.4, 0.0)$	$(1.5, 0.4, 0.0)$	$(3.6, 0.6, 0.0)$	$(2.0, 0.4, 0.2)$	$(5.3, 0.6, 0.2)$	$(9.5, 0.6, 0.4)$	Weibull
$(3.5, 0.2, 0.0)$	0.00	0.09	2.91	0.51	3.23	0.15	3.18	2.86	9.52
$(0.9, 0.2, 0.0)$	0.06	0.00	3.11	0.77	3.11	0.04	3.06	2.73	10.18
$(11.0, 0.4, 0.0)$	2.93	3.19	0.00	2.63	1.62	3.23	1.62	1.83	9.87
$(1.5, 0.4, 0.0)$	0.61	1.00	3.10	0.00	2.30	1.20	2.21	1.97	9.43
$(3.6, 0.6, 0.0)$	2.80	2.86	1.93	2.01	0.00	2.71	0.00	0.03	9.97
$(2.0, 0.4, 0.2)$	0.13	0.05	3.37	0.97	3.15	0.00	3.09	2.73	9.70
$(5.3, 0.6, 0.2)$	2.73	2.80	1.94	1.93	0.00	2.64	0.00	0.02	9.96
$(9.5, 0.6, 0.4)$	2.40	2.48	2.00	1.65	0.03	2.35	0.02	0.00	9.95
Weibull	44.50	45.43	71.65	45.22	37.00	17.78	33.87	29.77	0.00

Losses (in %) in D-efficiency for crossed comparison between the optimal design find for the Weibull model as reported in [Kim and Flournoy (2015)] and our models assuming various asymmetries through different values of $(\tilde{\alpha}_1, \tilde{\alpha}_2, \tilde{\alpha}_3)$.
